# Comparative Evaluation of Thyroid Profiles and Heart Rate Variability in Newly Diagnosed Subclinical Hypothyroid and Euthyroid Pregnant Women

**DOI:** 10.7759/cureus.98316

**Published:** 2025-12-02

**Authors:** Anchal Singh, Samir K Singh, Hanjabam Barun Sharma, Mamta Singh

**Affiliations:** 1 Physiology, Institute of Medical Sciences, Banaras Hindu University, Varanasi, IND; 2 Obstetrics and Gynaecology, Institute of Medical Sciences, Banaras Hindu University, Varanasi, IND

**Keywords:** autonomic regulation, heart rate variability, pregnancy, second trimester, subclinical hypothyroidism

## Abstract

Background

Subclinical hypothyroidism (SCH) is a common pregnancy condition that is frequently regarded as a biochemical finding with few functional implications. Thyroid hormones, however, have an impact on autonomic modulation, and even slight insufficiencies can change a mother's cardiovascular flexibility. Although heart rate variability (HRV) is a sensitive indicator of autonomic balance, there is little information available on HRV in pregnant women with SCH.

Methods

An analytical observational study was carried out on 41 pregnant women with recently diagnosed SCH and 40 pregnant controls who were euthyroid and in their second trimester. Lead II configuration resting five-minute ECGs were captured. LabChart Pro v8.1.30 software (AD Instruments, Castle Hill, Australia) was used to analyze them for HRV. Measurements were made in the frequency domain (low frequency (LF), high frequency (HF), LF/HF ratio, and total power), time domain (average duration of all normal-to-normal (NN) intervals between consecutive heartbeats (mean RR), standard deviation of normal-to-normal intervals (SDNN), root mean square of the standard deviation of interbeat intervals (RMSSD), and percentage of interbeat intervals varying by more than 50 ms (pNN50)), and nonlinear indices (standard deviation of the instantaneous beat-to-beat variability (SD1), standard deviation of the continuous long-term variability of RR intervals (SD2), and index of the balance between short-term and long-term variability (SD1/SD2)). With significance set at p<0.05, statistical analysis was conducted using either the Mann-Whitney U test or the unpaired t-test, depending on the distribution of the data.

Results

Mean RR was comparable between groups (p=0.331). Time-domain parameters were significantly lower in SCH: SDNN (p=0.005), RMSSD (p=0.035), and pNN50 (p=0.026). In the frequency domain, LF was significantly higher in SCH (p=0.04), whereas HF (p=0.281) and LF/HF ratio (p=0.143) were comparable. Total power was significantly reduced in SCH (p=0.0001). Nonlinear indices also showed reductions in SD1 (p=0.009) and SD2 (p=0.0001), while SD1/SD2 ratio remained comparable (p=0.585). Correlation analysis revealed no significant association between thyroid-stimulating hormone (TSH) and HRV indices (all p>0.05).

Conclusion

Subclinical hypothyroidism in pregnancy, though biochemically mild, is associated with early autonomic dysfunction characterized by reduced parasympathetic activity and diminished global HRV. These changes occur even in the absence of alterations in basal heart rate, suggesting subtle but functionally relevant cardiovascular impairment. HRV analysis may serve as a sensitive non-invasive tool for detecting early autonomic dysregulation in SCH and highlights the need for closer maternal surveillance during pregnancy.

## Introduction

Thyroid hormones are essential to both the physiology of the mother and the fetus throughout pregnancy. Pregnancy induces marked alterations in thyroid function due to several factors, including the stimulatory effect of human chorionic gonadotropin (hCG) on the thyroid gland, enhanced peripheral metabolism of thyroid hormones, and elevated levels of thyroid-binding globulin [1]. The frequency and clinical consequences of overt and subclinical thyroid dysfunction in expectant mothers have consequently grown to be a significant public health issue. It affects approximately 0.3% to 1.9% of pregnancies as overt hypothyroidism and 1.5% to 5% as subclinical hypothyroidism [2]. Maternal hypothyroidism prevalence in India has been shown to range from 1.2% to 67.0% [3]. Subclinical hypothyroidism (SCH) is typically defined by elevated serum thyroid-stimulating hormone (TSH) with normal free thyroxine (fT4) [4]. Subclinical hypothyroidism (SCH), even when clinically silent, has been associated with several adverse obstetric and neonatal outcomes, including miscarriage, preterm birth, gestational hypertension, intrauterine growth restriction, low birth weight, stillbirth, and impaired neurocognitive development in offspring [4-6]. Thyroid hormones exert direct and indirect effects on the cardiovascular system affecting lipid metabolism, heart rate, systemic vascular resistance, and cardiac contractility at the cellular level. Furthermore, they change the autonomic nervous system's equilibrium: hyperthyroid conditions frequently raise sympathetic activity and lower parasympathetic tone, whereas hypothyroid conditions are associated with a relative vagal predominance but might result in complex autonomic dysfunction [7].

Heart rate variability (HRV) measures beat-to-beat variability and offers information on sympathetic-parasympathetic balance [8]. It is a non-invasive indicator of autonomic nervous system modulation of the heart. Decreased heart rate variability (HRV) is a proven indicator of autonomic dysfunction and is linked to poor cardiovascular outcomes in both pregnant and non-pregnant individuals [9,10].

In order to meet the metabolic needs of both the mother and the baby, pregnancy itself causes autonomic adaptations, such as a rise in heart rate and blood volume, a decrease in systemic vascular resistance, and a change in sympathovagal balance [11]. Autonomic regulation may be further disrupted by endocrine disorders that are superimposed, such as subclinical hypothyroidism. A number of non-pregnancy studies show that both overt and subclinical thyroid problems change HRV parameters, indicating a higher risk for cardiovascular disease [12]. Literature review indicates that there are relatively few studies evaluating HRV in women with early or recently diagnosed maternal SCH; hence, we aim to evaluate and compare heart rate variability (HRV) indices between women with newly diagnosed subclinical hypothyroidism (SCH) and euthyroid pregnant women in their second trimester, in order to detect early autonomic alterations associated with SCH.

## Materials and methods

This was an analytical observational study conducted at the Institute of Medical Sciences, Banaras Hindu University, Varanasi, India. Pregnant women in their second trimester, aged 18-35 years who were receiving antenatal care in the Department of Obstetrics and Gynaecology, were enrolled between July 2024 and July 2025. SCH was detected from routine antenatal thyroid function testing. Serum TSH was measured for all participants during the second-trimester visit. Women with elevated TSH values were subsequently advised to undergo free triiodothyronine (fT3) and fT4 testing. New cases of SCH in pregnancy were diagnosed as per the American Thyroid Association (ATA) 2011 Guidelines using normal fT4 levels and a serum TSH cutoff of 3.0 μIU/mL during the second trimester [13]. For detailed autonomic assessment, these participants were subsequently invited to the Cardio-Autonomic and Vascular Medicine Laboratory, Department of Physiology, where further evaluations were carried out. Pregnant women with morbid obesity, diabetic vasculopathy, any known vascular disorder (systemic or local), multiple pregnancy, any known case of renal disease, valvular heart disease, any autoimmune disorder and already diagnosed case of hypothyroid pregnancy and related medication were excluded from the study. The Institutional Ethics Committee, Institute of Medical Sciences, Banaras Hindu University, Varanasi, India, approved the study (No. Dean/2024/EC/7045, dated: February 28, 2024). Written informed consent was obtained from all pregnant women.

Data acquisition

Participants were placed in a supine posture following a 15-minute rest period, and all measurements were conducted in a temperature-controlled environment. All women were asked to abstain from tea and coffee for 24 hours, and a light meal was advised to be consumed two hours prior to the test. LabChart Pro v8.1.30 software (AD Instruments, Castle Hill, Australia) was used to obtain Lead II electrocardiogram (ECG) tracings. Disposable Ag-AgCl electrodes were applied for recording the standard bipolar limb Lead II ECG. The digital band pass filter featured a low cutoff frequency of 0.5 Hz and a high cutoff frequency of 35 Hz, while the ECG collection and sampling frequency was kept at 1 kHz.

Analysis of heart rate variability

In the event of a transition or adjustment effect, the first one to two minutes of each data segment were removed from the entire recording. LabChart Pro v8.1.30 (AD Instruments, Australia) was used to measure HRV. The software's integrated HRV analysis toolkit was used to evaluate the data for five minutes. Data was thoroughly examined visually to eliminate those that contained movement artifacts or ectopics. Time domain indices (standard deviation of normal-to-normal intervals (SDNN), root mean square of the standard deviation of interbeat intervals (RMSSD), and percentage of interbeat intervals varying by more than 50 ms (pNN50)), frequency‑domain analysis of HRV included the power of high‑frequency (HF), (0.15-0.40 Hz); low‑frequency (LF), (0.04-0.15 Hz); and very low‑frequency (VLF), (below 0.04 Hz) power ranges, and LF and HF were presented also in normalized units and as a ratio. Poincare plot analysis consisted of SD1 and SD2 which are standard deviation of RR interval along major and minor axis, respectively. Scatter index is expressed as ratio of SD1 to SD2 reflecting the non‑linear HRV.

Statistical analysis

Microsoft Excel (Microsoft Corporation, Redmond, WA, USA) was used to enter the data. Data was analyzed using SPSS Statistics software (version 20.0; IBM Corp., Armonk, NY, USA). Categorical data were displayed as numbers and percentages, and continuous variables were represented as mean ± standard deviation (SD) or median (interquartile range) depending on the distribution. The independent Student's t-test was employed for data that was normally distributed, and the Mann-Whitney U test was employed for non-parametric data , in order to compare continuous variables between two groups. When applicable, Chi-square test was used to assess categorical variables. Spearman's correlation coefficients were used to analyze the relationship between TSH and HRV indices. p value <0.05 was taken as statistically significant.

## Results

The study comprised 81 pregnant women, 41 of whom had recently been diagnosed with subclinical hypothyroidism (SCH group), and 40 of whom were pregnant controls who were euthyroid. The two groups were comparable in terms of mean age, height, gestational age, and blood pressure (p > 0.05), and there was no discernible difference in parity distribution (p = 0.504). However, body weight and BMI were significantly higher in the SCH group compared to controls (p < 0.05). TSH levels were found to be significantly higher in the hypothyroid group (p = 0.0001), while fT3 and fT4 values remained within the normal range in both groups as indicated in Table 1.

**Table 1:** Descriptive statistics of participants characteristics of the study population SCH: subclinical hypothyroidism, BMI: body mass index, BP: blood pressure, TSH: thyroid-stimulating hormone, fT4: free thyroxine, fT3: free triiodothyronine. Data are expressed as mean ± SD or median (IQ range). Between-group comparisons were performed using unpaired t-test or Mann-Whitney U test as appropriate. #Analyzed using Mann-Whitney U test. p values <0.05 are significant; **p<0.01, ***p<0.001.

Parameters		SCH pregnant (n=41)	Euthyroid pregnant (n=40)	p values
Age (years)		27.41 ± 2.83	27.18 ± 2.84	0.704
Height (cm)		154.91 ± 5.32	154.45 ± 6.22	0.719
Weight (kg)		60.10 ± 8.50	54.49 ± 7.73	0.003**
BMI (kg/m²)		25.02 ± 3.22	22.88 ± 3.17	0.003**
Gestational age (weeks)#		17 (15-20)	18 (15-20)	0.556
Parity	Nulliparous	60.98% (25)	52.50% (21)	0.504
	Primiparous	39.02% (16)	47.50% (19)	
BP (Systolic)#		124 (116-128)	122 (112-128)	0.383
BP (Diastolic)		74.68 ± 8.37	77.33 ± 7.95	0.149
TSH (µIU/mL)#		5.77 (4.60-7.70)	2.49 (1.68-2.80)	0.0001***
fT4 (ng/dL)		1.035 ± 0.19	1.059 ± 0.246	0.626
fT3 (pg/mL)#		2.890 (2.503-3.295)	2.690 (2.363-3.308)	0.912

The comparison of time-domain, frequency-domain, and Poincare plot analysis of heart rate variability is shown in Table 2. Average duration of all normal-to-normal (NN) intervals between consecutive heartbeats (mean RR) was comparable between the group. However, there was a significant reduction in SDNN, RMSSD, and pNN50 in subclinical hypothyroid pregnant as compared to euthyroid pregnancy (p<0.05). LF (normalized unit (n.u)) was significantly higher in the subclinical hypothyroid group (p=0.04), while total power was significantly reduced (p<0.0001). HF (n.u) and LF/HF ratio were comparable between groups. Poincare plot analysis of HRV demonstrated a significant reduction in SD1 and SD2 in the subclinical hypothyroid group compared with the euthyroid group (p<0.05). However, the SD1/SD2 ratio was comparable between groups.

**Table 2:** Descriptive statistics of heart rate variability indices SCH: subclinical hypothyroidism, mean RR: average duration of all normal-to-normal (NN) intervals between consecutive heartbeats, SDNN: standard deviation of normal to normal interval, RMSSD: the square root of the mean of squares of the differences between adjacent NN intervals, pNN50: the proportion derived by dividing NN50 by the total number of NN interval, LF (n.u): low frequency in normalized unit, HF (n.u): high frequency in normalized unit, LF/HF ratio: index of sympathovagal balance, TP: total power (overall variance in HRV signal), SD1: standard deviation of the instantaneous beat-to-beat variability, SD2: standard deviation of the continuous long-term variability of RR intervals, SD1/SD2 ratio: index of the balance between short-term and long-term variability. Data are expressed as mean ± SD or median (IQ range). Between-group comparisons were performed using unpaired t-test or Mann-Whitney U test as appropriate. #Analyzed using Mann-Whitney U test. p values <0.05 are significant *p<0.05; **p<0.01, ***p<0.001.

Parameters	SCH pregnant (n=41)	Euthyroid pregnant (n=40)	p values
Mean RR (ms)	706.85 ± 93.19	727.67 ± 98.34	0.331
SDNN (ms)#	30.67 (21.64-44.89)	37.37 (31.14-51.05)	0.005**
RMSSD (ms)#	25.33 (17.47-37.36)	31.72 (22.20-52.93)	0.035*
pNN50 (%)#	3.83 (0.33-9.11)	7.52 (1.33-24.30)	0.026*
LF (n.u)	48.20 ± 16.10	40.26 ± 18.15	0.04*
HF (n.u)	51.40 ± 15.55	55.28 ± 16.56	0.281
LF/HF ratio#	0.95 (0.56-1.49)	0.77 (0.46-1.18)	0.143
Total power (ms^2^)#	620.10 (314.05-1050.75)	1500 (1053.50-2562.50)	0.0001***
SD1 (ms)#	15.05 (11.89-25.80)	22.46 (15.71-37.87)	0.009**
SD2 (ms)#	32.90 (26.05-49.58)	49.58 (39.75-64.04)	0.0001***
SD1/SD2 ratio#	0.48 (0.30-0.63)	0.49 (0.34-0.69)	0.585

Correlation analysis between TSH and HRV parameters revealed no significant associations. Specifically, TSH showed a weak, non-significant negative correlation with SDNN (r=- 0.71, p=0.658), HF (r=-0.47, p=0.769), and LF (r=-0.33, p=0.835), while a weak positive but non-significant correlation was observed with RMSSD (r=+0.113, p=0.481) (p>0.05 for all) (Table 3).

**Table 3:** Correlation analysis of TSH with heart rate variability indices in SCH pregnant r=correlation coefficient; p<0.05 considered statistically significant; Spearman’s correlation used for non-parametric variables. TSH: thyroid-stimulating hormone, SCH: subclinical hypothyroidism, SDNN: standard deviation of normal-to-normal intervals, RMSSD: root mean square of the standard deviation of interbeat intervals, HF: high frequency, LF: low frequency.

Parameter	r value	p value
TSH vs. SDNN	-0.71	0.658
TSH vs. RMSSD	+0.113	0.481
TSH vs. HF	-0.47	0.769
TSH vs. LF	-0.33	0.835

## Discussion

In the present study, we evaluated heart rate variability (HRV) in newly diagnosed subclinical hypothyroid pregnant women and compared it with euthyroid pregnant controls in their second trimester. We specifically included women in the second trimester, as this period is considered physiologically more stable compared to the first and third trimesters. Thyroid function and autonomic measurements may be complicated by the early rise in hCG hormone during gestation and the increase in hemodynamic strain in late pregnancy. Garg et al. [14] documented that the significant changes in autonomic function occur in the second trimester, and their finding of lower baroreflex sensitivity at this stage suggests that mid-pregnancy may be a time when the autonomic system is more vulnerable. Another study by Solanki et al. [9] reported HRV is reduced in pregnancy when compared to non-pregnant, and the second trimester showed a major decline compared to the first and third. Likewise, Soldin et al. [15] documented that for most thyroid function indices, the second trimester shows greater stability than the first or third. Hence, evaluating HRV during the second trimester allows for a more reliable assessment of the impact of subclinical hypothyroidism on autonomic modulation.

Our results revealed that the mean RR interval, a surrogate for basal heart rate, did not differ significantly between the SCH and euthyroid groups. This finding suggests that early thyroid failure may not yet exert a measurable influence on sinus node pacing. In overt hypothyroidism, bradycardia and prolonged RR intervals are well-recognized features, largely attributed to reduced β-adrenergic receptor density and diminished responsiveness to catecholamines. However, in subclinical hypothyroidism, circulating free thyroid hormone levels typically remain within reference ranges, and the degree of receptor downregulation may not be sufficient to slow intrinsic pacemaker activity. As a result, chronotropic competence at rest appears preserved, even though subtle autonomic changes are already detectable through HRV indices. Significant variations were observed in HRV indices that represent parasympathetic activation. The SCH group showed much lower time-domain metrics like SDNN, RMSSD, and pNN50, which suggests less global variability and vagal regulation. Thyroid hormones normally enhance vagal activity via regulation of acetylcholine release and maintain baroreflex sensitivity. In euthyroid pregnancies, this supports higher beat-to-beat variability and stable cardiovascular control. Our findings therefore suggest that even a modest thyroid hormone deficiency during pregnancy can suppress parasympathetic tone. This aligns with earlier reports by Yildiz et al. [16] that overt hypothyroidism results in a decrease in time-domain indices, which measure beat-to-beat vagal modulation. However, Sahin et al. [17] found no significant difference in time- or frequency-domain HRV measures compared with controls in non-pregnant patients. Our findings add to this knowledge by showing that a moderate thyroid hormone shortage during pregnancy can effectively inhibit parasympathetic activation. Such impairment might be a factor in SCH mothers decreased cardiovascular adaptation.

Frequency-domain analysis provided further insights. Subclinical hypothyroid pregnant women had considerably higher LF power in the frequency-domain analysis than controls, although HF power was comparable. Pattern reflects a compensatory increase in sympathetic drive to offset impaired β-adrenergic sensitivity [18]. Galetta et al. [19] similarly documented an increase in LF/HF ratio in subclinical hypothyroid patients, reflecting higher sympathetic activity, along with reduced HRV indices consistent with vagal withdrawal. These results align with our observation of attenuated vagal modulation, although the shift toward sympathetic dominance in our cohort was less pronounced, possibly because normal pregnancy itself increases sympathetic tone, thereby blunting group differences. Furthermore, in subclinical hypothyroid group, total power was significantly lower, which supports the finding of general autonomic dysregulation. Nonetheless, total power which is a measure of overall autonomic variability was significantly lower in the SCH group, reflecting generalized autonomic dysregulation. Such reductions in total variability signal impaired autonomic flexibility, which has been linked to cardiovascular risk in both thyroid disease and pregnancy-related complications.

Nonlinear HRV indices (SD1 and SD2) were considerably lower in women with SCH, suggesting a decrease in both short- and long-term heart rate variability. The euthyroid group, on the other hand, had greater SD1 and SD2 values, indicating overall improved autonomic flexibility. Despite the general decrease in variability, the SD1/SD2 ratio was comparable between groups, indicating that the balance between short- and long-term variability was maintained.

Our findings are consistent with previous studies in overt hypothyroidism, which have consistently reported reduced HRV indices reflecting diminished parasympathetic activity and increased sympathetic drive. Mahajan et al. [12] demonstrated abnormalities in both sympathetic function and decreased parasympathetic reactivity in subclinical as well as overt hypothyroid patients. The complicated interaction between pregnancy-related autonomic adaptations and subclinical thyroid dysfunction may be the reason why our group did not exhibit any significant changes in HF or LF/HF ratio. Pregnancy itself is characterized by increased sympathetic tone and altered vagal modulation, which may mask some of the expected group differences.

In our study, women with subclinical hypothyroidism had significantly lower HRV indices than euthyroid controls, but there was no apparent correlation between these changes and TSH levels. This is in contrast to some non-pregnant SCH studies, such as Galetta et al. [19], who reported inverse correlations between TSH and SDNN and a positive correlation with LF/HF ratio, suggesting that higher TSH levels may reflect greater autonomic imbalance. However, another report by de Miranda et al. [20] have also failed to demonstrate such linear associations, highlighting heterogeneity across populations. In fact, women with higher TSH values did not necessarily demonstrate greater autonomic impairment, suggesting that elevated TSH alone may not be the driving factor behind altered cardiac autonomic regulation. Therefore, the lack of correlation with TSH indicates that autonomic impairment is caused by a multifactorial interaction of subtle thyroid hormone insufficiency, receptor level changes in β-adrenergic signaling, and gestational cardiovascular adaptations rather than a simple linear relationship with pituitary TSH, even though SCH pregnancy is clearly associated with reduced HRV.

Thyroid hormones have a crucial role in preserving autonomic cardiovascular homeostasis through both central and peripheral mechanisms. They increase the myocardium's β-adrenergic receptor density and sensitivity, improve catecholamine production and turnover, and promote downstream signaling pathways that preserve cardiac chronotropy and contractility. Furthermore, the baroreceptor reflex arc's integrity is maintained by thyroid hormones, which guarantee quick parasympathetic heart rate modulation in response to changes in blood pressure. In subclinical hypothyroidism, although circulating free thyroid hormones often remain within the reference range, even subtle reductions at the tissue level can disrupt these finely tuned processes. Vagal output is attenuated by diminished baroreceptor sensitivity, while cardiac adaptation to sympathetic stimulation is decreased by blunted β-adrenergic responsiveness. A decrease in parasympathetic tone and a relative shift toward sympathetic predominance are the outcomes of these changes taken together. Heart rate variability indices measure this autonomic imbalance; reduced RMSSD, pNN50, and SDNN indicate vagal withdrawal and decreased overall variability, even when basal heart rate is maintained.

Pregnancy, a condition that already requires dynamic autonomic control, may be made more susceptible by such early changes. Further, reduced HRV has been clinically linked to poor cardiovascular outcomes, such as hypertension, arrhythmias, and an elevated risk of cardiovascular disease in later life. Changes in HRV during pregnancy have been connected to issues including gestational hypertension and preeclampsia [21]. The observed decrease in HRV in pregnant women with subclinical hypothyroidism may suggest a greater susceptibility to such issues, despite the fact that our study did not explicitly evaluate outcomes. This emphasizes the significance of early detection and careful monitoring of this population.

The strengths of our study include the focus on newly diagnosed subclinical hypothyroid pregnant women, a group that has not been extensively studied with respect to HRV. Standardized HRV analysis allowed for reliable comparisons between groups. However, certain limitations should be acknowledged. Our study was single centered with a modest sample size, which may limit the generalizability of the findings. HRV was assessed using short-term recordings, long-term Holter-based analysis may have provided additional insights. Furthermore, the absence of significant correlation between TSH and HRV indices suggests that other factors, such as duration of thyroid dysfunction, pregnancy-related hormonal influences may play an important role.

## Conclusions

Our results show that subclinical hypothyroidism (SCH) is linked to detectable changes in autonomic cardiovascular control, even though it is often asymptomatic and regarded as a modest biochemical anomaly. According to this, SCH is not clinically silent during pregnancy, as is commonly believed. Our cohort's lower parasympathetic indices (e.g., RMSSD, pNN50, SD1) and decreased overall variability (total power, SDNN) imply that a mild thyroid hormone shortage has an impact on the autonomic nervous system after the fact, especially on vagal modulation. Significantly, these abnormalities were identified even when there was no obvious bradycardia or significant variations in basal heart rate, underscoring the sensitivity of HRV as a functional measure at an early stage. In order to identify pregnant women with SCH who are more likely to experience cardiovascular maladaptation, gestational hypertension, or worse neonatal outcomes, early detection of HRV anomalies may be helpful. These findings further justify the possibility of longer-term autonomic function monitoring during pregnancy, prompt thyroid hormone replacement medication initiation, or tighter maternal supervision. Our findings emphasize the potential of HRV as a sensitive, non-invasive biomarker to help close the gap between functional cardiovascular evaluation and biochemical diagnosis in this susceptible population.
